# Nursing Staff Knowledge on the Use of Intraosseous Vascular Access in Out-Of-Hospital Emergencies

**DOI:** 10.3390/ijerph20032175

**Published:** 2023-01-25

**Authors:** Macarena Ibarra Romero, Juan Carlos Sánchez-García, Emanuele Cavazzoli, María Isabel Tovar-Gálvez, Jonathan Cortés-Martín, Nazaret Martínez-Heredia, Raquel Rodríguez-Blanque

**Affiliations:** 1 Hospital Universitario Clínico San Cecilio, 18016 Granada, Spain; 2 Research Group CTS1068, Andalusia Research Plan, Junta de Andalucía, 18071 Granada, Spain; 3 School of Nursing, Faculty of Health Sciences, University of Granada, 18071 Granada, Spain; 4 School of Nursing Ceuta Campus, Faculty of Health Sciences, University of Granada, 51001 Ceuta, Spain; 5 Departamento Pedagogía, Facultad de Ciencias de la Educación, Universidad de Granada, Campus de Cartuja s/n., 18071 Granada, Spain; 6 San Cecilio Clinical Hospital, 18071 Granada, Spain

**Keywords:** education, nursing, vascular access, emergency medicine, intraosseous device, intravascular

## Abstract

In healthcare practice, there may be critically injured patients in whom catheterisation of a peripheral venous access is not possible. In these cases, intraosseous access may be the preferred technique, using an intraosseous vascular access device (IOVA). Such devices can be used for infusion or administration of drugs in the same way as other catheterisations, which improves emergency care times, as it is a procedure that can be performed in seconds to a minute. The aim of this study was to analyse the level of knowledge of nursing staff working in emergency departments regarding the management of the intraosseous vascular access devices. To this end, a cross-sectional online study was carried out using an anonymous questionnaire administered to all professionals working in emergency and critical care units (ECCUs) in Granada district (Spain). The results show that 60% of the participants believe that with the knowledge they have, they would not be able to perform intraosseous vascular access, and 74% of the participants believe that the low use of this device is due to insufficient training. The obtained results suggest that the intraosseous access route, although it is a safe and quick way of achieving venous access in critical situations, is considered a secondary form of access because the knowledge of emergency and critical care professionals is insufficient, given the totality of the participants demanding more training in the management of intraosseous access devices. Therefore, the implementation of theoretical/practical training programmes related to intraosseous access (IO) could promote the continuous training of nurses working in ECCUs, in addition to improving the quality of care in emergency and critical care situations.

## 1. Introduction

One of the functions of nurses when a patient requires intravenous administration of fluids and medicines is the catheterisation of an effective venous access that allows for their administration. Occasionally—especially in out-of-hospital emergencies—there may be patients in a clinical situation in which it is very difficult to catheterise a peripheral access venous access; in such situations, intraosseous vascular access (IOVA) may be performed [1]. Patients may be in a state of haemodynamic compromise, e.g., during cardiopulmonary resuscitation without intravenous access in the first two minutes of resuscitation or in patients experiencing severe circulatory collapse caused by dehydration or diabetic ketoacidosis, drug intoxication, coma or shock [2,3].

IO provides access to the venous plexus within the bone marrow space, enabling drug administration in a similar pattern to that provided by peripheral venous access in both paediatric and adult populations [4,5]. The location of insertion is determined primarily by the patient’s age and condition, although it should be noted that insertion is acceptable for all age groups [2].

The American Heart Association (AHA) and the European Resuscitation Council (ERC) recommend the insertion of IOVA devices to reduce drug and fluid administration time during resuscitation and emergencies [4,6,7]. These devices have a higher first-time success rate, faster insertion times and lower complication rates compared to peripheral intravenous catheters [4,8], with insertion times varying from a few seconds to one minute and first-catheter insertion success rates of up to 97%. Through this IV, not only fluids and medications recommended for intravenous infusion but also blood and blood products, including marrow aspirate for venous blood gas analysis, can be administered [3,9,10]. Several IOVA devices are available, and brands and availability differ by institution [2,3,4], all of which provide fast access with few associated complications in emergency situations [11,12].

In terms of insertion sites, the literature suggests that the ideal site for IVA insertion is a long bone with easy reference points, e.g., the tibia or humerus, considering that access in the proximal humerus may be more suitable for improved flow rates, better drug administration, less pain and fewer complications [2,4,13,14]. It is necessary to consider the limitation of insertion time, as the recommended duration of placement for IO access devices is 24 h, only exceeding this time by an additional 24 h in cases in which alternative vascular access is not available; however, insertion time can never exceed a total of 48 h [2,4].

Contraindications for IO insertion include fractures of the bone of election or above the insertion site due to inadequate anatomical location, previous surgery involving the bone of election, infection at the site, local vascular compromise, compartment syndrome as a consequence of perforation through the posterior cortex, common soft tissue complications similar to venous infiltration, fluid extravasation, fat and air embolism, cellulitis, osteomyelitis, inability to remove a flexed IO cannula (may require surgical removal) and previous access at the selected anatomical location [2,4]. Another complication to consider is insertion failure associated with obesity [11].

Many benefits have been reported for the use of IOVA in critically ill patients, but there are some barriers to its use, one of which is the perception that nursing staff are unfamiliar with this technique [2]. However, few studies have been carried out in Spain on the practical knowledge of nursing staff in comparison with other countries in Europe or North America. Some such studies that have been realised in Spain provide positive data on the use of IVA, which means that it is necessary to produce more studies that support the analysis of nearby centres in order to improve morbidity and mortality in emergency situations [15].

Therefore, the general objective of this study is to analyse the level of knowledge of nursing staff working in Emergency and Critical Care Units (ECCUs) in the Granada district regarding the management of intraosseous vascular access. We also aims to analyse the level of training received in this subject, as well as the requirements perceived by these professionals.

## 2. Materials and Methods

### 2.1. Design

This study was carried out as an online cross-sectional study. The study was designed according to the Strengthening Reporting of Observational Studies in Epidemiology (STROBE) guidelines.

### 2.2. Objectives

The aim of this study was to analyse the level of knowledge of nursing staff working in emergency departments regarding the management of intraosseous vascular access devices.

### 2.3. Setting and Sample

The study participants are nurses working in ECCU services of the Granada Health District.

All nurses working in these services, a total of 73, distributed in the province of Granada, were invited to participate.

A non-probabilistic convenience sample was used in this study. No exclusion criteria were defined in the selection of the sample, as all nurses working in the ECCU services of Granada district were included, regardless of age, sex, year of finishing their nursing degree, type of employment contract or professional experience.

### 2.4. Ethical Considerations

This research conforms to the ethical requirements of the University of Granada. Prior to the interview, participants received instructions and an informed consent form, which included information about the aim of the study, process, method of data collection, rights, data handling and expected benefits of the research. The privacy of the participants and the place and time of the interviews was respected, and the participants were not identified.

Our article is presented in accordance with the guidelines of the University of Granada. The informed consent model and the page that specifies the ethics guidelines of the University of Granada can be accessed at https://www.ugr.es/universidad/normativa/normativa-comision-etica-investigacion (accessed on 11 September 2021).

All nurses who agreed to participate were accepted.

The data were treated confidentially and a questionnaire was sent to the nurses using a form hosted on the google drive website (URL; doc.google.com), which had contained personal information about the participants.

Before starting the questionnaire, participants were required to read and approve the informed consent form explaining the aim of the research. If they did not approve the informed consent, they could not continue to fill in the questionnaire.

There were no incentives to participate in the questionnaire, nor were there any reprisals for not participating in the study.

The study was approved by the Management of the Granada Metropolitan District Clinical Management Unit on 15 April 2021.

The research was conducted in accordance with the ethics standards of the Declaration of Helsinki [16].

### 2.5. Data Collection

The questionnaire was prepared by the researchers using a form from Google (Alphabet, Mountain View, CA, USA). Participant mail was obtained through the nursing care coordinator of the Granada District Emergency Department Clinical Management Unit, who granted approval for its use in accordance with the law on personal data protection.

Data were collected from October 2021 to June 2022.

### 2.6. Instruments

The questionnaire collected sociodemographic variables age, sex, year of completion of nursing studies, type of work contract, general professional experience and professional experience in out-of-hospital emergency services. The questions included in the questionnaire focused on general questions about participant training in the use of the device of interest, whether they had used it in their clinical practice or would like to receive training in this regard, as well as specific questions about the intraosseous vascular access device (Appendix A).

### 2.7. Statistical Analysis

Statistical analysis was performed using IBM SPSS software version 25.0 for Windows. All data were categorised by variables and expressed as frequencies and percentages, with means and standard deviations obtained. The chi-square test was used to compare the results (percentages) for each of the questions. Significance was accepted as *p* < 0.05.

The Instrument was validated by a panel of 7 experts including medical personnel and nursing specialists working in emergency and disaster services, applying statistical data on the degree of concordance, indicating an acceptable content validity based on significant concordance in expert judgment (*p* < 0.05).

The reliability of the questionnaire was determined to be 0.71 according to Cronbach’s alpha.

## 3. Results

Of the 73 nurses working in the ECCU of the Granada health district at the time of data collection, 50 professionals responded to the questionnaire. A proportion of 58% of the participants were women compared to 42% men, with ages ranging between 23 and 63 years (*p* = 0.074).

In terms of type of contract, 38% of the participants were civil servants, 22% were interim staff and 40% were on temporary contracts.

A proportion of 82% of the participants had more than 5 years of professional experience, and 18% had between 1 and 5 years of experience (*p* < 0.001). In terms of professional experience in out-of-hospital emergency services, 48% had more than 5 years of experience, 32% had between 1 and 5 years of experience and 20% had 0 to 1 years of professional experience.

With regard to the training received by the nursing staff, the mean was 1.34 ± 0.479. A proportion of 66% reported having received training in the use of an intraosseous vascular device, whereas 33% reported having received no training (*p* = 0.009). A proportion of 84% of the professionals reported not having used intraosseous vascular access compared to 16% who reported that they had (*p* = 0 < 0.001). A proportion of 94% said they thought they might encounter emergencies in which it would be necessary to use this route, whereas 6% did not think they would encounter such a situation (*p* < 0.001).

A proportion of 40% of the participants believed that with the knowledge they possess, they would be able to use perform vascular access, whereas 60% believed that they would not be able to use perform such a task (*p* = 0.08). A proportion of 74% of the participants believed that the limited use of this device is due to insufficient training, compared to 26% who did not believe that the lack of use is due to this insufficient training (*p* = 0.009).

All participants answered that they would like to receive more training on the insertion, care and maintenance of an intraosseous line.

In terms of specific knowledge of IO, 14% (*p* < 0.001) of participants answered correctly when asked about the anatomical location of first choice in adults when inserting an intraosseous vascular access, with 62% thinking that the anatomical location of choice in adults is the distal tibia (*p* < 0.001). When asked about the anatomical location of choice in paediatrics for the insertion of an intraosseous vascular access, 30% (*p* = 0.06) answered proximal tibia (the correct answer), whereas the remaining participants opted for other answers.

A proportion of 74% of the participants answered correctly when asked about checking the correct placement of an intraosseous catheter.

A proportion of 88% of the participants knew the answer to the question about substances that can be infused through such a device.

With regard to the question on how long it is advisable to keep an intraosseous vascular access in situ, 48% answered correctly. Finally, 66% answered correctly to the question about the complications that can be caused by the intraosseous route.

## 4. Discussion

The training of nursing professionals in the use of intraosseous access in life-threatening emergency situations is essential for the effective use of this venous access route. The strength of this study lies in the fact that the results indicate a lack of knowledge regarding this kind of vascular access, as shown in other similar studies [17,18]. A previous study reported that in a brief theoretical/practical training, almost all participants were able to proceed with the catheterisation of this kind of access [19]. The complications associated with the use of intraosseous devices described above can be reduced by appropriate training of staff before the use of the equipment and performance of the procedure [20]. It should be noted that in emergency situations, catheterisation is a priority, so if first-choice intravenous access cannot be achieved, IO access is a quick and safe alternative [2]. Moreover, given that the success rate of IO is twice as high as that of IV placement in critically ill trauma patients, its use is underestimated, as studies recognise that IO access can be obtained in 20 s, allowing for fast access in patients who may have difficulty with IV access [2,3].

A high percentage of participants indicated that they had not received training in the use of IO. Most of the professionals who participated in this study believe that they could encounter situations in which the use of the intraosseous route would be necessary; however, the majority believe that they would not be able to use it with the knowledge they have. However, in a similar study conducted in the emergency medical service (EMS) of Catalonia, the authors concluded that the majority of professionals had received extensive training and had a high degree of experience, which allows them to handle the IOVA correctly, although they suggested recycling, as not all professionals had a good level of knowledge [17]. Few articles published in Spain have assessed the knowledge of nursing staff in relation to the management of IO; therefore, additional such studies are necessary, specifically in Spain, as, in contrast to other countries, nurses are generally responsible for this procedure. All participants indicated a desire for more training in the management and care of IO, so it is necessary to include both theory and practical training programmes to increase the knowledge and skills of professionals in this technique, as was reported in similar studies [18] conducted in other countries; cases in which training has been carried out are associated with an increase in both the success of catheterisation and the level of confidence in performing the technique, as well as an increase in the level of knowledge regarding the control of complications [21,22,23,24]. Although in Spain, nurses and medical staff are the only personnel authorised to perform catheterisation, training is recommended for all healthcare professionals involved in emergency situations to provide adequate support [2].

Research on IOVAs has been carried out in emergency settings, so it is suggested that future studies be carried out on the implementation of this kind of device in other healthcare settings, as the documented speed and accuracy of insertion make this type of catheterisation a reliable alternative when classic routes are not possible [25]. A limitation of the current study is that it was carried out in a specific context, i.e., emergency departments in primary care, so the result presented herein may not be applicable to other out-of-hospital emergency nurses in other services.

## 5. Conclusions

This study reflects the need for theoretical/practical training of nursing staff in the management of intraosseous vascular access (IOVA), with the recommendation that it be implemented both among emergency personnel and in other services. It is also necessary to implement more extensive training plans starting in university studies and, in the case of emergency services, for these training programmes to be conducted for all personnel involved.

## Data Availability

Data regarding the study are available upon request to the corresponding author.

## Appendix A

Intraosseous Vascular Access Nursing Knowledge Assessment Questionnaire

Sociodemographic Data

- Age: •  Sex:  M F
- Year of completion of nursing studies:
- Type of contract: Civil servant Interim Eventual
- Professional experience: 0–1 year 1–5 years more than 5 years
- Professional experience in out-of-hospital emergency services:
  0–1 year 1–5 years more than 5 years

General Information

- Have you been trained in intraosseous route management?
  Yes No
- Have you used this type of vascular access in your clinical practice?
  Yes No
- Do you think you might encounter clinical emergencies where it would be necessary to use this type of vascular access?
  Yes No
- Do you think that, with your knowledge of intraosseous line management, you would be able to use this type of vascular access in an out-of-hospital emergency?
  Yes No
- Do you think that the low use of this device is due to insufficient training?
  Yes No
- Would you like to be trained in the insertion, care and maintenance of the intraosseous line?
  Yes No

Specific Knowledge

- Which anatomical location in adults would be the first choice when inserting an intraosseous vascular access?
  - Proximal humerus
  - Distal tibia
  - Distal femur
  - None of the above
- Which anatomical location in a paediatric patient would be the first choice when inserting an intraosseous vascular access?
  - Proximal tibia
  - Internal tibial malleolus
  - Distal humerus
  - A and B are correct
- How would you check the correct placement of the intraosseous catheter?
  - The needle must be stationary and fixed
  - Bone marrow aspiration with 10 mL syringe (not always obtained)
  - Liquid infusion without resistance
  - All are correct
- What substances can be infused through this device?
  - Colloids only
  - Crystalloids only
  - Any type of drug/liquid normally infused by I.V. route
  - All are false
- How long is it advisable to keep the intraosseous vascular access in situ?
  - Between 24 and 48 h
  - Up to 24 h
  - 48 to 72 h
  - No option is correct
- Which of these options would be a complication(s) of the intraosseous route?
  - Compartment syndrome
  - Extravasation
  - A and B are correct
  - A and B are false
